# Volunteer motivation in a CITiZAN community archaeology project at Sandwich Bay, Kent

**DOI:** 10.1080/20518196.2025.2517962

**Published:** 2025-07-16

**Authors:** Grace Conium Parsonage, Lara Band, Eleanor Williams

**Affiliations:** a Archaeology Department, Canterbury Christ Church University, Canterbury, UK; b Museum of London Archaeology, London, UK; c Independent Researcher

**Keywords:** Public, coastal, citizen science, longevity, evaluation, intertidal, qualitative data

## Abstract

Volunteer participation produces an invaluable wealth of support and data for the coastal and estuarine archaeology of the UK, which may otherwise go unrecorded, but what drives volunteers to conduct this work? Our paper aims to identify volunteer motivation for participating in fieldwork at Sandwich Bay, Kent, in February 2022, run by the Coastal and Intertidal Zone Archaeological Network (CITiZAN). Adopting methods within rapid ethnographic assessment (REA), which uses semi-structured interviews and participant observation, demonstrates that reasons for volunteering are numerous and varied. These include experiences, social factors, fear of losing archaeological knowledge, learning, and health and wellbeing. By better understanding volunteer motivations, projects in community archaeology can be better placed to recruit and retain their volunteers, where REA is demonstrated as an effective method for gaining this information.

## Introduction

Volunteers who participate in coastal citizen science initiatives and community archaeology through recording threatened archaeology provide a vital service in recording data that would otherwise be lost to climate change and coastal erosion (Dawson et al. 2013, 2020; Dawson, Hambly, and Graham 2017). Working with volunteers to gather data was witnessed before the terms community archaeology and citizen science were coined in the 1970s and 1990s respectively (Irwin 1995; Marshall 2002). For example, in ornithology, volunteers in the USA have been collecting data since 1900 as part of the Christmas Bird Count (Sullivan et al. 2009). People are therefore willing to donate their time and energy to serve scientific and environmental monitoring. But *why* do people continue to give their time to these causes?

What motivates volunteers within citizen science and community archaeology is a growing area of research (e.g. Bruyere and Rappe 2007; Kowalczyk 2016; Mankowski, Slater, and Slater 2011; Measham and Barnett 2008; Rotman et al. 2012; Weston et al. 2003; Wright et al. 2015). With the increasing reliance on volunteers within these fields attributed to the rising fear of anthropogenic climate change, reductions in funding, and staff cutbacks (Conrad and Hilchey 2011), the question of what motivates volunteers is heightened. More informed insight could encourage long-term, sustainable volunteer participation (Rotman et al. 2012) and promote project longevity (Ryan, Kaplan, and Grese 2001). If projects are unable to identify *why* participants take part, they leave themselves vulnerable to volunteer burnout and reduced participation (Measham and Barnett 2008, 549), which in turn could impact the overall success of the project.

Although volunteer motivation within citizen science and community archaeology has been considered, few studies have focused on volunteer motivation within coastal and intertidal environments (Johnston 2019), despite their unique settings, and the diverse opportunities and challenges they present. Motivation is sometimes considered within internal evaluation reports required by external funders, such as the National Lottery Heritage Fund (NLHF), which includes CITiZAN; unfortunately, this information is not automatically publicly available. Some have suggested this has led to a gap in the literature regarding the formal evaluation of archaeological public engagement projects (Applejuice Consultants 2008; Ellenberger and Richardson 2018; King 2016; Neal 2015). Therefore, our paper aims to address the academic gap by considering the motivations of a group of volunteers working as part of a Coastal and Intertidal Zone Archaeological Network (CITiZAN) programme, whilst also presenting an effective evaluative approach for a short-term project of this nature.

To address this gap in academic literature, and to investigate the motivations of CITiZAN volunteers at this event, data from the study for the doctoral thesis of the first will be used. Data for the research was gathered, analysed, and written for our article by Grace Conium Parsonage, with supporting information on the project's background provided and written by Lara Band, one of the Project Officers for CITiZAN. Band was also the PhD supervisor of Conium Parsonage along with Ellie Williams. Williams supported editing our article; Band and Williams supported Conium Parsonage (henceforward ‘I/ my’) throughout the PhD.

My research is based on a qualitative approach using ethnographic principles carried out during the penultimate session of fieldwork at Sandwich Bay, Kent, in February 2022, for *(Don’t let it) Slip through the net*, a project run by the CITiZAN from 2018 to 2022 (see Band and Cvetković 2022; Conium 2022). I will apply methods within rapid ethnographic assessment (REA) (Sangaramoorthy and Kroeger 2020) using semi-structured interviews and participant observation. It will demonstrate that my methodology is a viable and effective way to evaluate community archaeology projects, including gathering information on volunteer motivation which can be applicable to a range of land and waterscape environments, adding to the growing body of works which have utilized REA within community archaeology (Jones 2017; Zaina, Proserpio, and Scazzosi 2021). It will also highlight the multifaced motivations of volunteers within a community archaeology event at the coast, and demonstrate to project leads and organizations (e.g. CITiZAN), that people’s reasons for attending can transcend a desire to engage with the archaeology itself. By considering these various motivations, projects may open doors to recruiting other strands of volunteers that may not have been considered. In addition, through using REA to gain a more nuanced understanding of individual and group motivation, projects can better position themselves to ensure continued and productive volunteer support.

### Background to CITiZAN

CITiZAN was an NLHF-supported citizen science project focusing on coastal archaeology and climate change. The project was embedded within the Museum of London Archaeology (MOLA) and ran from 2015 to 2018 and 2019 to 2022, with six regional ‘Discovery Programmes’ established for the second phase. The East Kent Coast Discovery Programme, led by archaeologist/project officer Lara Band, focused on the project *(Don’t let it) Slip through the net* at Sandwich Bay (see Band and Cvetković 2022). Over nine fieldwork sessions from 2019 to 2022, 24 volunteers helped record, sample, research, and report on the remains of post-medieval ‘keddle nets’, a type of V or bow shaped static fish trap with nets hung on wooden poles. Support and further funding from Historic England enabled the sampling, with volunteers, of the remains of keddle net poles and the subsequent wood analysis (Demicoli and Damian 2022).

Beyond the significance of the archaeological investigation itself, *(Don’t let it) slip through the net* was designed to impact future methodologies for community orientated projects and align closely with Historic England’s heritage strategy. Investigating this previously unrecognized site helped answer a Historic England Coastal, Marine and Maritime Heritage Research Question (2021b): *How can we mobilise volunteers and community groups to help assess the significance of identified historic wrecks, and coastal or submerged archaeological sites?*

In actively involving volunteers in the design of research questions, fieldwork, documentary research, and reporting, Band’s aim for *(Don’t let it) slip through the net* was to create a holistic understanding of the archaeological process. In helping participants develop skills, confidence, knowledge and enhanced heritage literacy to take with them beyond the life of the project (see Frearson 2018; Hedge and Nash 2016; Thomas 2010, 30), the project also aimed to help *build [community] capacity in engaging and cost effective ways* (Historic England 2021a: Strategic Aim 05) and *help people to make unique memories in the historic environment through participative experiences* (Historic England 2021a. Strategic Aim 08.2).

Band also designed the project as a response to Historic England’s call to consider the interrelationship of historic and natural environments (Historic England 2020). A knowledge exchange with Kent Wildlife Trust volunteers at East Blean managed woodland was to highlight the wider historical, geographical and material context of the keddle nets, explore the interlinked nature of cultural and natural heritage and provide an opportunity to discuss climate related changes, threats and mitigation strategies across different interest communities. An ‘open weekend’ for the public was also planned, including talks and walks by biogeographer Peter Vujakovic, Kent Wildlife Trust and Sandwich Bay Bird Observatory Trust. Unfortunately, plans for both events were unable to progress due to the uncertainty of the COVID-19 pandemic. In April 2022, however, CITiZAN and volunteers were able to visit East Blean woods in the company and conversation of Kent Wildlife Trust Ranger John Wilson and MOLA specialists Marvin Demicoli (archaeobotany) and Damian Goodburn (timber and woodwork) who also presented the preliminary results of the wood analysis (Demicoli and Damian 2022). That *(Don’t let it) slip through the net* was selected by Historic England as a best practice case study for their e-learning programme indicates the project achieved its aims.

### Motivation within citizen science: previous research

Researchers in Psychology have studied volunteer motivation for several decades (e.g. Clary and Snyder 1999). Yet, authors in citizen science (e.g. Bruyere and Rappe 2007; Ryan, Kaplan, and Grese 2001; Viduka and Edney 2022; Weston et al. 2003) and community archaeology (e.g. Kowalczyk 2016) argue it is understudied within their respective fields. Literature that considers general motivations of volunteer participation in community archaeology has been published in the USA (Kowalczyk 2016) and the UK (Frearson 2018; Roberts 2016), demonstrating the increasing recognition of the topic's importance. Within citizen science, participant motivation has been evaluated for projects in Africa (Wright et al. 2015), Australia (Weston et al. 2003), and the USA (Bruyere and Rappe 2007; Mankowski, Slater, and Slater 2011; Rotman et al. 2012). For heritage at intertidal sites, further published considerations have been made by the Thames Discovery Programme in the UK (Johnston 2019) and Gathering Information via Recreational and Technical (GIRT) Scientific Divers initiatives in Australia and New Zealand (Viduka and Edney 2022).

A range of motivations has been identified within citizen science. Measham and Barnett (2008) identified six themes of volunteer motivation projects in natural resource management. These are: contributing to the community, social interaction, personal development, learning about the environment, a general ethic of care for the environment, and an attachment to a particular place. Viduka and Edney (2022) note the top five motivations of GIRT scientific divers as seeing underwater plant and animal life, enjoying the peace of the underwater environment, gaining an appreciation of nature, seeing new things, and having a sense of discovery. However, it is important to consider that motivations are not static. Frearson (2018) states that reasons why people volunteer in archaeology within the UK differ depending on demographics; mental and physical health were reasons for people in older age groups volunteering, and skill development and employability are more common reasons for those under 50. This recognition of volunteer motivation as subject to change over time is also noted elsewhere (Rotman et al. 2012; Ryan, Kaplan, and Grese 2001). Therefore, approaches and methodologies need to adapt and reflect this diversity.

Our paper presents a qualitative approach to capturing the motivations for volunteer participation within a community archaeology/ citizen science setting. An REA followed by a Thematic Analysis was used, revealing valuable insights into volunteer motivation and views on project impact and legacy, which will be considered further elsewhere.

## Method

From Saturday 19 February to Sunday 20 February 2022, the eighth of nine fieldwork sessions of *(Don’t let it) slip through the net* was organized at Sandwich Bay, Kent, under the direction of Lara Band. The aim was to continue recording post-medieval fish trap remains in the intertidal zone, and to take samples for wood analysis. Volunteers present ranged from those who had been with CITiZAN for several years, some with experience of a few fieldwork sessions and some entirely new to the project and archaeological volunteering. I joined intending to identify people’s motivations for participating, and ideas surrounding project impact and legacy.

### Rapid ethnographic assessment (REA)

REA allows project evaluation to take place within a narrow time frame (Sangaramoorthy and Kroeger 2020). Unlike traditional ethnography which uses a more inductive approach, REA compiles research questions formulated ahead of the data gathering. This acts as a guide for the approach and questions to ask participants. Conium Parsonage’s approach was ideally suited to the CITiZAN project at Sandwich Bay, as the group meets sporadically over short periods, usually several days at a time. The methods, therefore, needed to be quickly applied to gather as much data as possible in a limited time frame. Despite the limited time, employing a range of data collection techniques – semi-structured interviews, fieldnotes, and a reflective diary, and applying those to the set of predetermined research questions  – it was possible to conduct a successful REA of a *(Don’t let it) slip through the net* fieldwork session.

To capture both emic (insider view, in this case, the volunteers) and an etic (external view, in this case, the researchers) perspectives, participant observation and volunteer interviews were carried out. Using an REA framework requires the research questions to be set ahead of time. Therefore, a framework of questions was used to guide the interviews, which centred on their engagement and reasons for attending, how they thought participation benefited them, and their understanding of the legacy of the project, where the latter is considered further in Conium Parsonage. The volunteer interviews were semi-structured, conducted during fieldwork, and recorded using a handheld recorder away from the main group to ensure anonymity. I then transcribed each interview. The interviews were considered together with participant observation, recorded through fieldnotes recorded manually onsite, as well as offsite in a reflective field diary (see Bernard 2017). I observed the group with consent and made notes on actions of behaviour or selected quotes of speech when they aligned with the types of questions asked during the interviews. Once all the data from the interviews and participant observation had been collected and transcribed, it was analysed.

### Data analysis

Once the recordings were transcribed, I assigned each participant a pseudonym to ensure anonymity. I conducted eight semi-structured interviews with all volunteers on site. From the transcriptions, I analysed the data using thematic analysis (Braun and Clarke 2006, 2019). At this stage, it is important to note some external factors that directly influenced both fieldwork and the data collection. The original event was due to take place over three days (18th to 20th February 2022). However, at this time, a series of storms affected the country, including the east Kent coast. As a result, the Band took the difficult decision to cancel on Friday 18th February. In addition, the remaining dates were cold with high winds. This impacted the recordings as I shortened some interviews due to the wind; the voices were sometimes muffled, and the wind chill meant that participants wanted to keep active.

## Results

Once I transcribed and analysed the interviews using thematic analysis for all eight volunteers, several themes relating to why they engaged in the fieldwork from the 19th to the 20th of February 2022 at Sandwich Bay emerged. Reasons for volunteering were diverse and grouped into experience, social factors, fear, learning, and health and wellbeing. The rest of this section explores these themes in greater detail.

### Participants

**Figure 1.**
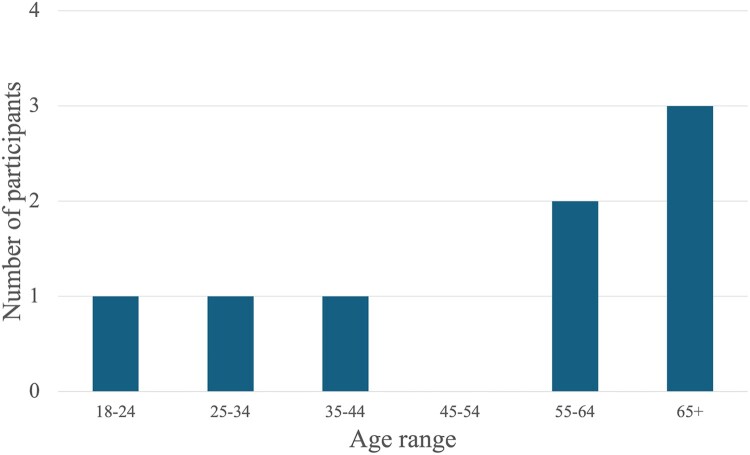
Table of age ranges of participants at (Don't let it) slip through the net on 19th and 20th February 2022, Sandwich Bay.

Five of the eight project volunteers present during the project were male, and three were female. There was also a diverse set of age ranges, as presented in Figure 1. While it was not within the scope of my investigation to collect all the demographic information relating to the volunteers, some were collected on the basis that certain characteristics, such as age, may influence the motivation of participation (Frearson 2018). Due to the small number of participants, no other factors will be presented here to ensure the anonymity of those taking part. Trends concerning motivations related to age will be presented where appropriate. However, these are only intended as observations, as further research with greater participant numbers would be needed to make direct inferences concerning age and motivation. They feature here to suggest the multifaced reasons for engagement are not static and will shift throughout the life of a volunteer.

### Motivation as experience

**Figure 2.**
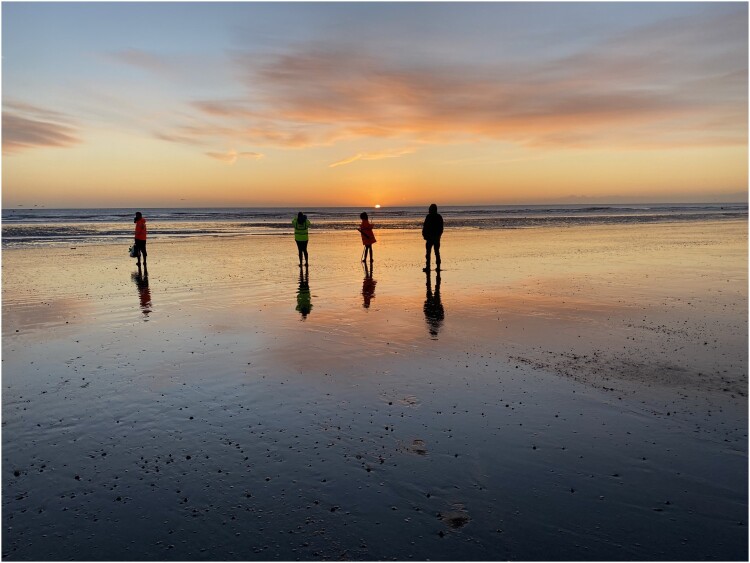
Volunteers watching the sunrise at Sandwich Bay on Saturday 19th February 2022. Taken by Grace Conium Parsonage.

Motivation as experience had the widest range of motivations for volunteers. Volunteers mentioned having a sense of discovery, novel experiences, and experiencing nature as reasons for engaging. Experiencing nature was a key motivator for participating in the *(Don’t let it) slip through the net* event. A particular draw mentioned by ‘Adam’ and the Lead Archaeologist at the start of the session is the chance to watch the sunrise at Sandwich Bay. The opportunity to watch the sunrise is also featured in publicity materials and event recruitment. Because the ‘best’ (i.e. lowest) tides at Sandwich Bay invariably fall early in the morning, the group often met before dawn and could watch the sunrise on the foreshore. On the first day of the event, the sun rose as the group was walking out to the foreshore to find the fish trap remains. The whole group stopped for a few moments to watch in almost silence. It was a mindful moment to start the day and could be considered a ‘reward’ for the early start. It is not only the sunrise that people noted as a motivation for experiencing nature. The beach environment itself was another draw for the volunteers. Being on the beach at a low tide, with only a few anglers, bait diggers, and dog walkers, was a novel experience for some, where one likened the environment to the surface of the moon. The natural environment motivated volunteers to join and engage with the project, not just the historic environment and archaeological significance. Of those who mentioned the natural environment, there is an even spread across the age ranges, as six of the eight volunteers mention its significance (Figure 2).

Adam: “Getting up early in the morning and seeing the sunrise on the beach.”

Alfred: “I was particularly attracted to doing things and coming out to the beaches and the foreshore … ”

### Motivation as social

Social factors were also a motivator for people to join in and meet others. Volunteers are aware of the benefits of these communal gatherings. Here, people can engage with those with similar interests to their own, as well as those they ordinarily may not socialize with in their everyday lives. Also noted was a sense of disappointment that the overall project was finishing, and the group would no longer have a shared sense of purpose to continue meeting up. Sadness and even anxiety anticipating the eventual end of a community project have been noted elsewhere (Hardy and Williams 2019). Projects such as this provide the opportunity and structure that allow people to meet and socialize in particular – often unique – environments and with a range of others. The age ranges of the people citing social reasons for attending these sessions were diverse, highlighting the importance placed on social interaction across a person's lifetime.

Arnold: “But I also like working with others as well. Part of the reason, having moved out of this part of the world about 5 years ago is to have that [social] activity.”

Adam: “Because I'm down here every six months or so with the team, and we’ve got a nice group of us who get together, and I'll be sorry that we won’t be doing it. But I hope we do find something else to do.”

### Motivation as fear

Another motivation concerns ‘apocalyptic fears’. The notion of using apocalyptic language when referencing climate change within the humanities was observed by Neimanis, Åsberg, and Hedrén (2015) in their account of challenges within the humanities concerning the intersection of political, academic, and popular engagement with environmental issues. This theme is a reference to an understanding from the volunteers that the coastline is shifting, and archaeological features on the foreshore are in danger of disappearing due to climate change. This is, perhaps, unsurprising given the position of CITiZAN, which has a public-facing focus on archaeology disappearing due to coastal erosion. The ‘Who we are’ page, for example, states that the project *highlights the threat of coastal erosion to a wealth of foreshore and intertidal sites* where they *have established an infrastructure and network of volunteers with the skills, commitment and support to record, monitor and promote fragile and threatened archaeological intertidal sites* (CITiZAN 2021). Volunteers have also used this language when describing their motivations for attending, as they also see the archaeological features on the coast as at risk of disappearing due to the threats of climate change.

Andrea: “Archaeology is fascinating, it feels like we are doing something which is useful in terms of future climate change, trying to identify features on the foreshore, which could possibly become covered up in the following years.”

Angela: “Because the nature of the fact of them being wooden, they are disappearing. I mean, some of them aren’t actually visible today. So, there’s no record of these things and of this industry. It's just completely disappeared now.”

Six of the eight participants mentioned the issues of the disappearing coast or climate change impacting the coastline; the two that did not were both in the above 56 age category. Whilst this could indicate a greater awareness (or concern) of climate change issues in the younger volunteer groups, a lack of awareness cannot be implied for these two individuals, only that it was not mentioned during the interviews. Overall, the frequent citing of this signified a demonstratable awareness from the volunteers not only of the fragility of the remains that are being recorded, but of the entire site itself.

### Motivation as learning

Some volunteers came to learn more about their local heritage, or the field of archaeology. Alison wanted to know more about the industrial heritage of Sandwich, and the activity that used to happen on the foreshore. Antonio on the other hand was concerned with acquiring more knowledge and skills regarding archaeology, and how professionals conduct it at the coast. This difference in interests highlights the diversity of the wants and expectations of the volunteers. Different types of knowledge and skills motivate the individual volunteers within the group. The ability to understand these desires and address them during projects can influence the retention of existing volunteers. While it is impossible to adapt and tailor projects and events so that they cater to all volunteers’ motivations and expectations, awareness of these can lead to more meaningful engagement and increased participation, with obvious implications for the longevity and sustainability of projects. The age profiles of those citing learning as a motivation for attending were diverse, demonstrating the importance of learning across a person's lifetime.

Alison: “Well, I'm interested in archaeology, even though I don’t know much about it. And of course, I know Sandwich Bay well but I didn’t know anything about the fish traps so I thought it’s about time I knew something.”

Antonio: “To gain experience in archaeology, in coastal archaeology, because I have already experienced normal archaeology.”

### Motivation as health and wellbeing

Health and wellbeing was the final motivation discussed by the volunteers. They recognized the physical and mental wellbeing benefits of participating in coastal archaeology and quoted it as a direct influence on individual participation. Involvement in these types of citizen science projects allows volunteers to reap the health and wellbeing benefits of keeping physically active, with the mental stimulation of the project at hand. Both volunteers who cited health and wellbeing within this context were above 65 years old. Retirement specifically was mentioned by Arnold, where this project allowed him to remain mentally active.

Alfred: “I’ve always liked the active side of archaeology. Digging, or field surveys, or whatever.”

Arnold: “Because I'm in retirement, it’s just to keep my mind active.”

## Discussion

By using a qualitative methodology inspired by REA, an assessment of the *(Don’t let it) slip through the net* project run by CITiZAN has revealed a range of different motivations by the volunteers involved. Our method demonstrates not only similarities across respondents in their motivations to participate but also their nuance. Concerning the demographics of the volunteers, a few observations were made regarding their reasons for attendance. These included the importance in the older age categories placed on health and wellbeing, as well as the trend which saw younger volunteers citing archaeological loss through coastal erosion and climate change. While these are interesting observations, the participant sample size was small, so more work with larger groups needs to be carried out in these environments to determine how demographics influence motivation in citizen science and community archaeology.

Some of the motivations that arose from the data have been supported elsewhere. These include motivation concerning volunteer learning (e.g. Kowalczyk 2016; Power and Smyth 2016); social advantages (e.g. Ryan, Kaplan, and Grese 2001); health and wellbeing (e.g. Frearson 2018); and experiencing nature (Johnston 2019; Viduka and Edney 2022; Wright et al. 2015). The last is of particular interest to consider further, as two of the three examples citing experiencing nature as a motivation for participation were also intertidal sites (Johnston 2019; Viduka and Edney 2022).

Helen Johnston’s (2019) work recognizes volunteer motivation as experiencing the natural environment at the foreshore. Her work focuses on The Thames Discovery Programme (TDP), launched in 2008, working with the public to record archaeological features and finds from the foreshore of the Thames River. Johnston (2019) argues, based on formal evaluations and anecdotal conversations, that a key motivator for volunteer participation revolves around maintaining a positive relationship with the natural and historic environment of the Thames. Both CITiZAN and the TDP work with the public at intertidal sites. Within the interviews carried out here, it is seen that the Sandwich Bay foreshore was perceived as a unique natural environment by the CITiZAN volunteers who often spoke of the opportunity to watch the sunrise.

This is supported by the work of Viduka and Edney (2022), who also found that GIRT volunteer divers in Australia and New Zealand were also driven by factors relating to the coastal natural environment, including enjoying the peace and tranquillity of the underwater and intertidal setting. Coastal sites are drawing individuals to citizen science projects whose interests potentially lie outside the realm of heritage or the historic environment. It is possible to overlook this observation with the assumption that people are joining due to their interest in a site’s cultural remains and historic environment. The qualitative evaluation carried out at Sandwich Bay demonstrates that this is not necessarily the case, particularly in the context of intertidal sites, where the work of Johnston (2019) and Viduka and Edney (2022) supports this claim. This recognition has been used within CITiZAN, where the opportunity to watch the sunrise in a novel landscape was promoted by Lara Band. Our finding provides an opportunity for other similar projects at sites of natural significance, including intertidal sites, to take advantage of this knowledge, and find possible volunteers in a potentially unexplored demographic.

However, within these areas of coastal landscape, which can act as a motivation for participation, there exists the recognition amongst those working in these environments that it is changing. There is an acknowledgement within the current body of research that volunteer motivation within citizen science projects includes fear linked to helping the environment (Ryan, Kaplan, and Grese 2001). This recognition that coastal environments are under threat is supported by the reported motivations of volunteers at Sandwich Bay. Here, they have described the process of coastal erosion which ‘threatens’ archaeological features. Motivations driving people to volunteer stemming from a desire to conserve the natural environment is also found in other fields, such as conservation (Bruyere and Rappe 2007; Ryan, Kaplan, and Grese 2001; Weston et al. 2003). Therefore, the CITiZAN programme aligns with these motivations, which include volunteers working to conserve the natural environment.

The recording of archaeological sites and features at the coast is framed in the context of ‘threat’ from climate change by CITiZAN on their website. Venture et al. (2021) also make this point regarding the work of CITiZAN and other similar projects including Climate, Heritage and Environments of Reefs, Islands, and Headlands (CHERISH) in Wales and Ireland. In addition, Scotland’s Coastal Heritage at Risk Project (SCHARP) is another example where a project uses this type of language within their work, including within their name. However, this is not a trend limited to community archaeology and citizen science at the coast. In their discussion surrounding problems currently faced by the environmental humanities, Neimanis, Åsberg, and Hedrén (2015) have also stated that the current use of language in describing heritage and climate change is not productive. They argue this negative language does not lead to effective participation in environmental projects, and environmental dialogues require a more balanced tone to encourage more creative thinking around the topic, as well as a stronger sense of participation and involvement. Consideration of the language used to recruit and retain volunteers which uses a balanced tone, highlighting the possibilities for community archaeology to contribute to archaeological knowledge could help manage some of the fears experienced by those involved. Community archaeology projects, such as those run by CITiZAN, provide a platform to address these fears constructively; Conium Parsonage and Band Witnessed this at Sandwich Bay, which added to the information on post-medieval fish traps, which was recognized by those interviewed.

Within the interviews of the volunteers for the *(Don’t let it) Slip through the net* event in February 2022, the volunteers are aware of the consequences of climate change at the shoreline in Sandwich Bay. By engaging with the work of CITiZAN, they can actively contribute to the archaeological knowledge at this site. What could be considered an ‘inevitable loss’ has the potential to bring discoveries to the area (Venture et al. 2021), in this case regarding the post-medieval fishing industry of Sandwich Bay. Kerr (2023) has also recognized the ability of citizen science to potentially assist with greater climate communication and climate action with the use of deep maps of the coastal site near Doonanore Castle.

Other considerations of volunteer motivation within archaeology in Europe exist, and add to the narratives explored here. Boom (2018) reported that while there were many reasons for volunteers to participate in projects undertaken in the Netherlands, their strongest motivation for joining was their interest in history and archaeology. In Italy, four main categories of reasons behind engagement with archaeological projects were outlined as communication, participation, education, and support, where the former is the most common, highlighting the need for projects to keep local communities informed of ongoing projects (Ripanti 2022, 45). These differences highlight the changing motivations which can exist with volunteers across time and space, and highlight the need for greater attention in specific cases or locations. This is reinforced by the finding that motivations for volunteering are subject to change over time (Rotman et al. 2012)

Our work has demonstrated that the motivations for volunteer participation within community archaeology are multifaceted. REA provides a quick and inexpensive evaluation tool which can help those managing these projects to better understand these diverse drives. This can lead to further recruitment of a more diverse volunteer workforce, and better enable projects to retain those volunteers.

## Conclusion

By using a qualitative approach based on REA to assess a community archaeology project, some interesting themes regarding volunteer motivation of the *(Don’t let it) slip through the net* event have been revealed. My methodology characterized by REA, using pre-defined research questions, semi-structured interviews, and participant observation has provided rich data within a limited time frame. The methodology could, therefore, provide a useful evaluation tool for future citizen science and community archaeology projects to recruit and retain volunteers. This is particularly relevant for smaller, short-term projects that may have funding constraints.

My qualitative approach has demonstrated a range of motivations with the volunteers of the *(Don’t let it) slip through the net* fieldwork in February 2022. These were identified as:

- Experience: Volunteers were drawn to this event by its unique natural environment, including watching the sunrise and the coastal landscape. This factor was recognized by the Project Officer Lara Band and Conium Parsonage as a way of promoting the events at Sandwich Bay.
- Social factors: Volunteers recognized the social opportunities that taking part in the event provided. Meeting like-minded people and engaging in communal activities played a significant role in their participation
- Fear of archaeological knowledge loss: There was also an awareness amongst the volunteers concerning climate change and its impact on coastal archaeology. They recognized the threat of coastal erosion to archaeological features and felt compelled to contribute to preserving this heritage. By participating in this fieldwork, they could actively contribute towards the archaeological research conducted in Sandwich, which would have otherwise gone unrecorded if not for the efforts of CITiZAN.
- Learning: Some volunteers sought opportunities to learn more about their local heritage and archaeology, while others aimed to acquire new knowledge and skills in archaeology, enhancing their personal development.
- Health and wellbeing: Participation in these projects provided volunteers physical and mental health benefits. They appreciated staying physically active while being mentally stimulated by the archaeological work.

In the face of anthropogenic climate change, the focus on intertidal sites is crucial as they are at the forefront of its effects. Working with local communities and volunteers in these spaces offers a valuable opportunity to gather archaeological and historical information at these sites. Through projects such as CITiZAN, including the fieldwork opportunities at Sandwich Bay, volunteers can reconsider what is termed ‘inevitable loss’ (Venture et al. 2021), bringing discoveries to the East Coast and providing an invaluable wealth of support and data for the coastal and estuarine archaeology of the UK coastline which may otherwise go unrecorded.

## References

[CIT0001] Applejuice Consultants. 2008. *The Social Impact of Heritage Lottery Funded Projects. Evaluation Report on Research Conducted for Heritage Lottery Fund During 2006–2007.* Accessed November 8, 2022. https://www.heritagefund.org.uk/sites/default/files/media/research/socialimpacthlffunding.pdf.

[CIT0002] Band, Lara, and Tijana. Cvetković. 2022. “Part I – Fieldwork and research.” In *(Don’t Let It) Slip Through the Net: CITiZAN Investigations of Fish Traps at Sandwich Bay, Kent, 2018-2022*, edited by Lara Band, 1–114. London: MOLA (unpublished report, OASIS ID Molas1-509352).

[CIT0004] Bernard, H. Russell. 2017. *Research Methods in Anthropology: Quantitative and Qualitative Approaches*. 6th ed. London: Rowman & Littlefield.

[CIT0005] Boom, Krijn H. 2018. *Imprint of Action: the Sociocultural Impact of Public Activities in Archaeology*, 245. Leiden: Sidestone Press.

[CIT0006] Bruyere, Brett, and Silas Rappe. 2007. “Identifying the Motivations of Environmental Volunteers.” *Journal of Environmental Planning and Management* 50 (4): 503–516. 10.1080/09640560701402034.

[CIT0008] CITiZAN. 2021. “CITiZAN: Who We Are.” *CITiZAN*. Accessed April 5, 2022. https://citizan.org.uk/about-us/who-we-are/.

[CIT0009] Clary, E. Gil, and Mark Snyder. 1999. “The Motivations to Volunteer: Theoretical and Practical Considerations.” *Current Directions in Psychological Science* 8 (5): 156–159. 10.1111/1467-8721.00037.

[CIT0010] Conium, Grace. 2022. “Part IV - Ethnographic Evaluation.” In *(Don’t Let It) Slip Through the Net: CITiZAN Investigations of Fish Traps at Sandwich Bay, Kent, 2018-2022*, edited by Lara Band, 254–282. London: MOLA (unpublished report, OASIS ID Molas1-509352).

[CIT0011] Conrad, Cathy C., and Krista G. Hilchey. 2011. “A Review of Citizen Science and Community-based Environmental Monitoring: Issues and Opportunities.” *Environmental Monitoring and Assessment* 176 (1-4): 273–291. 10.1007/s10661-010-1582-5. 20640506

[CIT0012] Dawson, Tom, Joanna Hambly, and Ellie Graham. 2017. “A Central role for Communities: Climate Change and Coastal Heritage Management in Scotland.” In *Public Archaeology and Climate change*, edited by Tom Dawson, Counrtney Nimura, Elías López-Romero, and Marie-Yvane Daire, 23–33. Oxford: Oxbow Books.

[CIT0013] Dawson, Tom, Joanne Hambly, Alice Kelley, William Lees, and Sarah Miller. 2020. “Coastal Heritage, Global Climate Change, Public Engagement, and Citizen Science.” *Proceedings of the National Academy of Sciences* 117 (15): 8280–8286. 10.1073/pnas.1912246117. PMC7165427 32284415

[CIT0015] Dawson, Tom, Anna Vermehren, Alan Miller, Iain Oliver, and Sarah Kennedy. 2013. “Digitally Enhanced Community Rescue Archaeology.” In *Proceedings of 2013 Digital Heritage International Congress (DigitalHeritage)*, Marseille, France, 28 October–1 November 2013.

[CIT0016] Demicoli, Marvin, and Goodburn Damian. 2022. “Part II - Wood Analysis Report.” In *(Don’t Let It) Slip Through the Net: CITiZAN Investigations of Fish Traps at Sandwich Bay, Kent, 2018-2022*, edited by Lara Band, 159–228. London: MOLA (unpublished report, OASIS ID Molas1-509352).

[CIT0018] Ellenberger, Kate, and Lorna-Jane Richardson. 2018. “Reflecting on Evaluation in Public Archaeology.” *AP: Online Journal in Public Archaeology* 8 (1): 65–94. 10.23914/ap.v8i1.141.

[CIT0019] Frearson, Debbie. 2018. “Supporting Community Archaeology in the UK: Results of a 2018 Survey.” *CBA Research Bulletin* 6.

[CIT0021] Hardy, Lesley, and Eleanor Williams. 2019. “The People before Us’ Project: Exploring Heritage and Well-Being in a Rapidly Changing Seaside Town.” In *Historic Landscapes and Mental Well-being*, edited by Timothy Darvill, Kerry Barrass, Laura Drysdale, Venessa Heaslip, and Yvette Staelens, 215–227. Oxford: Archaeopress.

[CIT0023] Hedge, Rob, and Aisling. Nash. 2016. *Assessing the Value of Community-Generated Historic Environment Research*. Online at https://historicengland.org.uk/research/support-and-collaboration/research-frameworks-typologies/assessing-community-generated-research/.

[CIT0024] Historic England. 2020. “Heritage and the Environment.” Accessed March 5, 2021. https://historicengland.org.uk/content/heritage-counts/pub/2020/heritage-environment-2020/.

[CIT0025] Historic England. 2021a. “Corporate Plan 2021-2.” Accessed October 13, 2023. https://historicengland.org.uk/images-books/publications/he-corp-plan-2021-22/.

[CIT0026] Historic England. 2021b. “Coastal, Marine and Maritime Heritage Research Questions”. *Historic England*. Accessed January 11, 2022. https://historicengland.org.uk/research/current/discover-and-understand/coastal-andmarine/.

[CIT0027] Irwin, Alan. 1995. *Citizen Science: A study of People, Expertise and Sustainable Development*. Vol. 136. London: Routledge.

[CIT0028] Johnston, Helen. 2019. “Messing about on the River: Volunteering and Well-being on the Thames Foreshore.” In *Historic Landscapes and Mental Well-being*, edited by Timothy Darvill, Kerry Barrass, Laura Drysdale, Venessa Heaslip, and Yvette Staelens, 179–188. Oxford: Archaeopress.

[CIT0029] Jones, Siân. 2017. “Wrestling with the Social Value of Heritage: Problems, Dilemmas and Opportunities.” *Journal of Community Archaeology & Heritage* 4 (1): 21–37. 10.1080/20518196.2016.1193996.

[CIT0030] Kerr, Sarah. 2023. “Citizen Science and Deep Mapping for Climate Communication: A Report on CHICC.” *Journal of Community Archaeology & Heritage* 10 (2): 82–89. 10.1080/20518196.2022.2051139.

[CIT0031] King, Eleanor M. 2016. “Systematizing Public Education in Archaeology.” *Advances in Archaeological Practice: A Journal of the Society of American Archaeology* 4 (4): 415–424. 10.7183/2326-3768.4.4.415.

[CIT0032] Kowalczyk, Stefanie. 2016. “Excavating the “Who” and “Why” of Participation in a Public Archaeology Project.” *Advances in Archaeological Practice* 4 (4): 454–464. 10.7183/2326-3768.4.4.454.

[CIT0033] Mankowski, Trent A., Stephanie J. Slater, and Timothy F. Slater. 2011. “An Interpretive Study of Meanings Citizen Scientists make When Participating in Galaxy Zoo.” *Contemporary Issues in Education Research* 4 (4): 25–42.

[CIT0034] Marshall, Yvonne. 2002. “What Is Community Archaeology?” *World Archaeology* 34 (2): 211–219. 10.1080/0043824022000007062.

[CIT0037] Measham, Thomas G., and Guy B. Barnett. 2008. “Environmental Volunteering: Motivations, Modes and Outcomes.” *Australian Geographer* 39 (4): 537–552. 10.1080/00049180802419237

[CIT0038] Neal, Cath. 2015. “Know Your Place? Evaluating the Therapeutic Benefits of Engagement with Historic Landscapes.” *Cultural Trends* 24 (2): 133–142. 10.1080/09548963.2015.1031479.

[CIT0039] Neimanis, Astrida, Cecilia Åsberg, and Johan Hedrén. 2015. “Four Problems, Four Directions for Environmental Humanities: Toward Critical Posthumanities for the Anthropocene.” *Ethics and the Environment* 20 (1): 67–97. 10.2979/ethicsenviro.20.1.67.

[CIT0040] Power, Andrew, and Karen Smyth. 2016. “Heritage, Health and Place: The Legacies of Local Community-Based Heritage Conservation on Social Wellbeing.” *Health & Place* 39:160–167. 10.1016/j.healthplace.2016.04.005. 27126363

[CIT0042] Ripanti, Francesco. 2022. *Unforgettable Encounters: Understanding Participation in Italian Community Archaeology*. Oxford: Archaeopress Publishing Ltd.

[CIT0043] Roberts, Hayley. 2016. “The Practice of Community Archaeology in the UK: A Model for Best Practice Based upon Case Studies from Dorset and Cambridgeshire.” PhD diss., Bournemouth University.

[CIT0044] Rotman, Dana, Jenny Preece, Jen Hammock, Kezee Procita, Derek Hansen, Cynthia Parr, Darcy Lewis, and David Jacobs. 2012. “Dynamic Changes in Motivation in Collaborative Citizen-Science Projects.” In *the Proceedings of the ACM 2012 Conference on Computer Supported Cooperative Work*.

[CIT0045] Ryan, Robert L., Rachel Kaplan, and Robert E. Grese. 2001. “Predicting Volunteer Commitment in Environmental Stewardship Programmes.” *Journal of Environmental Planning and management* 44 (5): 629–648. 10.1080/09640560120079948.

[CIT0046] Sangaramoorthy, Thurka, and Karen A. Kroeger. 2020. *Rapid Ethnographic Assessments*. Milton: Routledge.

[CIT0047] Sullivan, Brian L., Christopher L. Wood, Marshall J. Iliff, Rick E. Bonney, Daniel Fink, and Steve Kelling. 2009. “eBird: A Citizen-Based Bird Observation Network in the Biological Sciences.” *Biological conservation* 142 (10): 2282–2292. 10.1016/j.biocon.2009.05.006.

[CIT0048] Thomas, Suzie. 2010. *Community Archaeology in the UK: Recent Findings*. York: Council for British Archaeology. Accessed February 20, 2019. https://tuhat.helsinki.fi/ws/portalfiles/portal/40303543/CBA_Report_2010.pdf.

[CIT0049] Venture, Tanya, Caitlin DeSilvey, Bryony Onciul, and Hannah Fluck. 2021. “Articulating Loss: A Thematic Framework for Understanding Coastal Heritage Transformations.” *The Historic Environment: Policy & Practice* 12 (3-4): 395–417. 10.1080/17567505.2021.1944567.

[CIT0050] Viduka, Andrew, and Joanne Edney. 2022. “GIRT Scientific Divers Citizen Science Programme: Volunteer Motivations and Characteristics.” *Journal of Community Archaeology & Heritage* 9 (3): 143–160. 10.1080/20518196.2021.2005411.

[CIT0051] Weston, Michael, Michael Fendley, Robyn Jewell, Mary Satchell, and Chris Tzaros. 2003. “Volunteers in Bird Conservation: Insights from the Australian Threatened Bird Network.” *Ecological Management & Restoration* 4 (3): 205–211. 10.1046/j.1442-8903.2003.00169.x.

[CIT0052] Wright, Dale R., Les G. Underhill, Matt Keene, and Andrew T. Knight. 2015. “Understanding the Motivations and Satisfactions of Volunteers to Improve the Effectiveness of Citizen Science Programs.” *Society & Natural Resources* 28 (9): 1013–1029. 10.1080/08941920.2015.1054976.

[CIT0053] Zaina, Federico, Licia. Proserpio, and Giulia. Scazzosi. 2021. “Local Voices on Heritage: Understanding Community Perceptions Towards Archaeological Sites in South Iraq.” *Journal of Community Archaeology & Heritage* 8 (4): 256–272. 10.1080/20518196.2021.1958615.

